# Demonstration of X-ray Thomson scattering as diagnostics for miscibility in warm dense matter

**DOI:** 10.1038/s41467-020-16426-y

**Published:** 2020-05-26

**Authors:** S. Frydrych, J. Vorberger, N. J. Hartley, A. K. Schuster, K. Ramakrishna, A. M. Saunders, T. van Driel, R. W. Falcone, L. B. Fletcher, E. Galtier, E. J. Gamboa, S. H. Glenzer, E. Granados, M. J. MacDonald, A. J. MacKinnon, E. E. McBride, I. Nam, P. Neumayer, A. Pak, K. Voigt, M. Roth, P. Sun, D. O. Gericke, T. Döppner, D. Kraus

**Affiliations:** 1 Lawrence Livermore National Laboratory, Livermore, CA 94550 USA; 2 Institut für Kernphysik, Technische Universität Darmstadt, Schlossgartenstraße 9, Darmstadt, 64289 Germany; 3 Helmholtz-Zentrum Dresden-Rossendorf, Bautzner Landstraße 400, Dresden, 01328 Germany; 4 SLAC National Accelerator Laboratory, 2575 Sand Hill Road, Menlo Park, CA 94025 USA; 5 Institute of Solid State and Materials Physics, Technische Universität Dresden, Dresden, 01069 Germany; 6 Department of Physics, University of California, Berkeley, CA 94720 USA; 7 Lawrence Berkeley National Laboratory, Berkeley, CA 94720 USA; 8 University of Michigan, Ann Arbor, MI 48109 USA; 9 European XFEL GmbH, Holzkoppel 4, Schenefeld, 22869 Germany; 10 GSI Helmholtzzentrum für Schwerionenforschung, Planckstraße 1, Darmstadt, 64291 Germany; 11 Centre for Fusion, Space and Astrophysics, Department of Physics, University of Warwick, Coventry, CV4 7AL United Kingdom

**Keywords:** Laboratory astrophysics, Astrophysical plasmas

## Abstract

The gas and ice giants in our solar system can be seen as a natural laboratory for the physics of highly compressed matter at temperatures up to thousands of kelvins. In turn, our understanding of their structure and evolution depends critically on our ability to model such matter. One key aspect is the miscibility of the elements in their interiors. Here, we demonstrate the feasibility of X-ray Thomson scattering to quantify the degree of species separation in a 1:1 carbon–hydrogen mixture at a pressure of ~150 GPa and a temperature of ~5000 K. Our measurements provide absolute values of the structure factor that encodes the microscopic arrangement of the particles. From these data, we find a lower limit of $2{4}_{-7}^{+6}$% of the carbon atoms forming isolated carbon clusters. In principle, this procedure can be employed for investigating the miscibility behaviour of any binary mixture at the high-pressure environment of planetary interiors, in particular, for non-crystalline samples where it is difficult to obtain conclusive results from X-ray diffraction. Moreover, this method will enable unprecedented measurements of mixing/demixing kinetics in dense plasma environments, e.g., induced by chemistry or hydrodynamic instabilities.

## Abstract (web summary)

It is challenging to reliably probe the miscibility behavior of elements in extreme conditions. Here, the authors use X-ray Thomson scattering and compare to the X-ray diffraction method in order to determine mixing of different atomic species in warm dense matter conditions.

## Introduction

The current state of the large bodies in our solar system is an essential benchmark for our understanding how planetary systems form and evolve. Modern planetary probes such as the Juno mission^1^ provide a growing wealth of data, such as magnetic field configurations^2^, gravitational moments^3^ and zonal winds^4^, even for distant planets like Jupiter. However, such data can only be obtained for the space outside the planets or their upper layers. To construct the internal structure and composition from these data, knowledge about the properties of the compressed matter deep inside a planet is required. In particular, a better understanding of high-pressure, high-temperature mixtures is of paramount importance for describing the internal structure of gas and ice giants, where gravity compresses matter into the 100 GPa to 10 TPa range. The high internal temperatures of these planets create an environment with thermal energies similar to those of chemical bonds and above. In the deeper layers, thermal excitations can thus trigger a complex series of material modifications and phase transitions, possibly including species separation and demixing^5^.

Miscibility-driven phase transitions in compressed matter are very important for planetary modeling for two reasons: (i) they may be significant enough to define layer boundaries prohibiting full convective mixing within the planet and, thus, strongly influence the mass distribution inside planets^6^ and (ii) heavier components created by demixing will liberate gravitational energy by precipitation and, thus, may strongly modify the energy balance of the planets. For gas planets like Jupiter and Saturn, a separation into helium-rich and helium-poor phases has been predicted for some region inside the planet and the potential presence of a corresponding boundary layer has a significant impact on the interpretation of observational data and our understanding of planetary formation and evolution^4,7–10^. On icy giants like Uranus and Neptune, water, methane and other hydrocarbons are highly abundant. Experimental evidence shows the dissociation and polymerization of methane under pressure, leading to a heavy hydrocarbon fluid^11,12^. Under certain conditions, diamond can form^13–15^ and will precipitate towards lower layers due to its high density.

The highly compressed matter inside large planets is in a complex state called warm dense matter covering densities comparable to solids and temperatures from several thousand to several hundred thousand kelvins. Although its study has gained significant momentum in recent years, it still holds many challenges for a theoretical description due to strongly interacting particles and the quantum nature of its electrons^16,17^. In particular, the complex interplay between the different components is extremely hard to model and basic mixing schemes generally fail to adequately represent the physics, although they are widely applied due to their simplicity^18^. To move beyond such simple models, dedicated laboratory experiments are needed to determine the properties of mixtures in this parameter regime.

Mixing and demixing processes in dense plasma environments also significantly impact state-of-the-art ICF capsule implosions^19,20^. In particular, hydrodynamic instabilities can result in ablator-fuel mix which prevents efficient compression and ignition of the fuel^21^. Furthermore, phase separation of carbon and hydrogen may be relevant when using CH plastic ablator materials. This phenomenon could lead to local density fluctuations and seed hydrodynamic instabilities^21,22^. Such instabilities, in particular ablation-front Rayleigh–Taylor growth, were identified as one of the primary issues that led to reduced implosion performance during the National Ignition Campaign, where the first shock of the implosion drive creates pressures of 100–200 GPa and temperatures of 6000–10,000 K in the ablator material^19^, i.e., conditions similar to the environment inside gas and ice giants.

In this article, we investigate the phase separation of hydrocarbons under warm dense matter conditions as they occur inside icy planets and during the first compression stages in ICF implosions using plastic ablators. Whereas previous analysis has established diamond formation in these samples with X-ray diffraction (XRD) from diamond crystallites^15,23^, here, we present a quantitative analysis of species separation applying X-Ray Thomson scattering (XRTS) that does not rely on the presence of crystalline structures. The theory of XRTS predicts strong sensitivity of the elastic scattering to different degrees of mixing^24^, while the inelastic scattering from free electrons can be used as a reliable diagnostics for the basic plasma parameters like density, temperature and ion charge state^25–27^. We now extend the applicability of XRTS and demonstrate the feasibility of a quantitative measurement of the degree of species separation in carbon-hydrogen mixtures.

## Results

### Experimental method

**Fig. 1:**
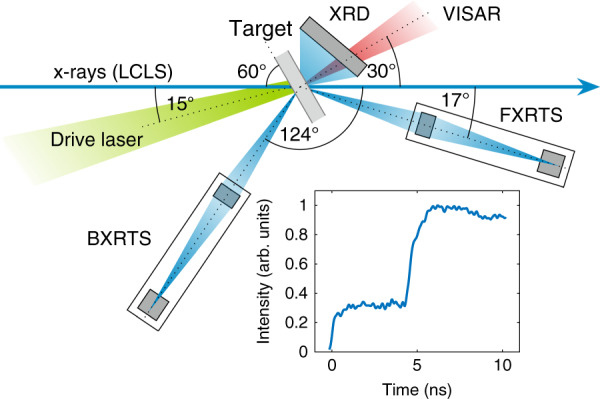
**Schematic of the experimental setup**. The drive laser compresses the sample using a stepped pulse profile (see inset). The C–H separation is recorded in situ using XRTS and XRD simultaneously. The shock dynamics are constrained by an optical Velocity Interferometer System for Any Reflector (VISAR).

**Fig. 2:**
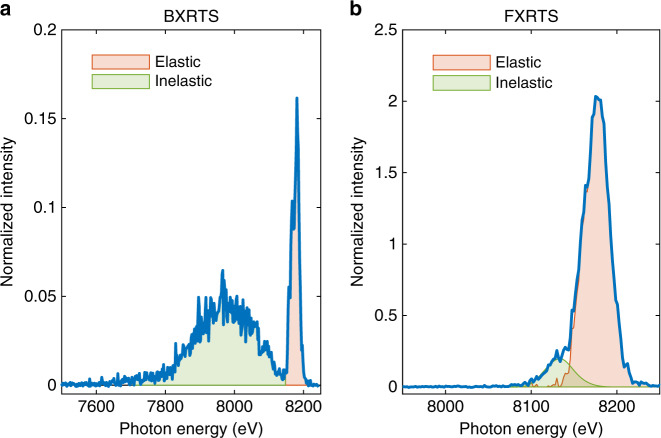
**XRTS spectra**. The scattered X-ray signals are recorded with the (**a**) backward and (**b**) forward spectrometers, at scattering angles of 123° and 17° to the incident X-rays, respectively. The integrated areas for Eqs. (2) and (3) are shaded in red and green, respectively. Both spectra have been corrected for energy-dependent influences and are normalized to the integrated inelastic scattering measured on the backward spectrometer.

A schematic of the experimental setup is shown in Fig. 1. Polystyrene (C_8_H_8_)_*n*_ samples were irradiated by two co-propagating high-energy laser beams forming a stepped pulse profile, resulting in two shock compression waves coalescing at the sample rear side. The compressed samples were probed in situ by X-rays with a photon energy of 8180 eV and a pulse duration of 50 fs. The scattered X-rays were detected by two spectrometers, one in backward scattering geometry (BXRTS, *θ*_b_ = 123°, *k* = 7.30 Å^−1^) and one in forward scattering geometry (FXRTS, *θ*_f_ = 17°, *k* = 1.23 Å^−1^). Examples of the recorded spectra are shown in Fig. 2. More details on the experimental setup can be found in the “Methods” section.

One-dimensional radiation hydrodynamic simulations were applied to estimate the thermodynamic conditions inside the compressed sample. We obtain a pressure of (60 ± 7) GPa and a temperature of (4000 ± 500) K in the first shock, whereas the second shock reaches (150 ± 15) GPa and (5000 ± 500) K. The quoted uncertainties are based on the observed gradients in time and space in the simulations^28^. More details can be found in the “Methods” section. These conditions are similar to those predicted at a depth of  ~10,000 km inside Uranus and Neptune^29,30^. Additionally, these results are in good agreement with XRD measurements during the same experiment^15^.

To measure the amount of species separation, we use X-ray Thomson scattering (XRTS). Here, the recorded scattering intensity is proportional to the total electron structure factor. In our experiment, we investigate temperatures well below 1 eV and thus, the contribution of free electrons can be neglected. Accordingly, the total electron structure factor for a multi-component system can be expressed as ref. ^24^

1 $$ S(k,\omega )=	\ {\mathop{\sum }\limits_{\alpha ,\beta }}\sqrt{{x}_{\alpha }{x}_{\beta }}\,\,{f}_{\alpha }(k){f}_{\beta }(k){S}_{\alpha \beta }(k,\omega )\\ 	+{\mathop{\sum }_{\alpha }}\,{Z}_{\alpha }^{{\rm{c}}}{x}_{\alpha }\int{\tilde{S}}_{\alpha }^{{\rm{ce}}}(k,\omega -\omega ^{\prime} ){S}_{\alpha }^{{\rm{s}}}(k,\omega ^{\prime} )\,\text{d}\omega ^{\prime} ,$$

where *x*_*i*_ = *n*_*i*_/∑_*α*_*n*_*α*_ is the concentration of the ion species *i*. For an isotropic system, all terms solely depend on the magnitude of the scattering vector ∣**k**∣ = *k*. The first term in Eq. (1) is referred to as weight of Rayleigh or elastic scattering *W*_R_. Here, it accounts for scattering from electrons that are bound to ions and thus dynamically follow their motion. The form of the bound state is given by the atomic form factor *f*_*α*_(*k*) for species *α*. The ionic microstructure is accounted for by the partial structure factors that can be treated statically *S*_*αβ*_(*k*, *ω*) = *S*_*αβ*_(*k*)*δ*(*ω*), since the ion motion cannot be resolved in the discussed experiment. The second term represents scattering inducing bound-free transitions. ${Z}_{\alpha }^{{\rm{c}}}$ bound core electrons of each species are described by the inelastic structure factor for bound electrons ${\tilde{S}}_{\alpha }^{{\rm{ce}}}(k,\omega )$ modulated by the self-motion of the ions ${S}_{\alpha }^{{\rm{s}}}(k,\omega )$.

**Fig. 3:**
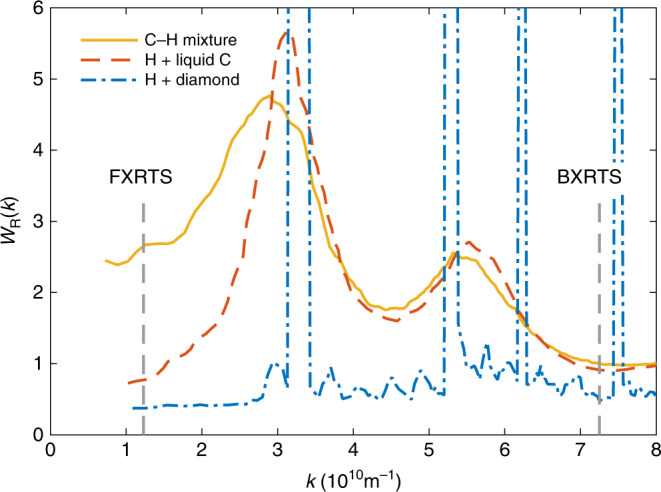
**DFT-MD simulations**. Structure calculations for *p* = 156 GPa and *T* = 5000 K show a significant drop in the Rayleigh scattering *W*_R_ for CH in a demixed state for *k* ≲  2.5 × 10^10^ m^−1^ when assuming either liquid carbon or diamond in the pure carbon phase. The dashed vertical lines mark the wavenumbers probed by the spectrometers.

In order to extract the degree of species separation, a minimum of two spectra are required; here, we have forward (FXRTS) and backward (BXRTS) scattering. The much better understood backward scattering provides the absolute calibration of the forward scattering signal, from which the degree of species separation can be inferred. Moreover, this scheme requires the inelastic contribution of the FXRTS to be known with high accuracy including any potential influence of condensed matter physics effects, e.g. originating from compressed diamond^31,32^. For these tasks, we apply quantum ab initio simulations using density functional theory and molecular dynamics (DFT-MD) for the Rayleigh scattering and time-dependent DFT together with a linear response formalism for the determination of the inelastic structure^33–39^ (for more details, see the “Methods” section). From DFT-MD, it is also possible to estimate the expected Rayleigh scattering^40^ *W*_R_(*k*) for pure carbon (in the liquid form or diamond), pure hydrogen as well as mixed and fully demixed CH. As shown in Fig. 3, the Rayleigh scattering is highly sensitive to demixing of the material compound at low values of *k*, which are probed by FXRTS.

For absolute calibration of the elastic scattering strength, we employ our backward scattering spectrum BXRTS. This quantity can be expressed by the intensities of elastic and inelastic scattering^41–43^:

2 $${W}_{{\rm{R}}}^{{\rm{b}}}(k)	={\mathop{\sum }_{\alpha ,\beta }}\sqrt{{x}_{\alpha }{x}_{\beta }}[{f}_{\alpha }(k){f}_{\beta }(k)]{S}_{\alpha \beta }(k) \\ 	={\mathop{\sum }_{\alpha }}\,{x}_{\alpha }\left[\mathop{\sum }\limits_{n = 1}^{{Z}_{\alpha }^{{\rm{wb}}}}\left(1-{f}_{n,\alpha }^{2}\left(k\right)\right)\right]\frac{{I}_{{\rm{el}}}^{{\rm{b}}}}{{I}_{{\rm{inel}}}^{{\rm{b}}}}.$$

Here, ${Z}_{\alpha }^{{\rm{wb}}}$ includes all weakly bound electrons of ion species *α* and *f*_*n*,*α*_(*k*) denote their atomic form factors of each electron^44^. *I*_el_ and *I*_inel_ are the spectrally integrated scattering intensities of the elastic and inelastic scattering. Eq. (2) is only dependent on the ratio of these two intensities and does not rely on the absolute inelastic scattering signal intensity. Therefore, it can be used to calculate *W*_R_(*k*) in backward direction. Here, the Compton shift *E*_C_ = 198 eV is large enough to clearly separate the elastic and inelastic scattering signals. At this energy, the electrons of the carbon K-shell with a binding energy significantly larger than *E*_C_ are neglected in the bound-free scattering for the analysis. Summing over all other more weakly bound electrons in Eq. (2) results in ${W}_{\mathrm{R}}^{\mathrm{b}}=2.50\frac{{I}_{{\rm{el}}}^{{\rm{b}}}}{{I}_{{\rm{inel}}}^{{\rm{b}}}}$ for a 1:1 mixture of hydrogen and carbon.

### Inferred C–H species separation

We are now able to process our data by normalizing the FXRTS data, that carries information about miscibility, to the BXRTS signal via

3 $$\frac{{W}_{{\rm{R}}}^{{\rm{f}}}}{{W}_{{\rm{R}}}^{{\rm{b}}}}=\frac{{\epsilon }^{{\rm{b}}}{I}_{{\rm{el}}}^{{\rm{f}}}}{{\epsilon }^{{\rm{f}}}{I}_{{\rm{el}}}^{{\rm{b}}}\cdot {\chi }_{{\rm{c}}}}.$$

The indices b and f denote the backward and forward spectrometer, respectively. The polarization factors^26^ for the two spectrometer geometries due to the horizontal polarization of the XFEL beam are given by *ϵ*^b^ = 0.320 and *ϵ*^f^ = 0.915. ${\chi }_{{\rm{c}}}=1.9{8}_{-0.11}^{+0.12}$ accounts for the relative difference in spectrometer and detector sensitivity which was obtained by recording Ni-K_*β*_ emission simultaneously with both spectrometers. ${I}_{{\rm{el}}}^{{\rm{b}}}$ and ${I}_{{\rm{el}}}^{{\rm{f}}}$ are the spectrally integrated elastic signals marked in Fig. 2. Due to the overlap of elastic and inelastic scattering on the forward spectrometer, the inelastic part has been modeled using TD-DFT simulations for the experimental conditions reached.

Using Eq. (3), the Rayleigh scattering in forward direction ${W}_{{\rm{R}}}^{{\rm{f}}}$ can now be calculated. Comparing this value to the DFT-MD simulations shown in Fig. 3, the Rayleigh scattering can be converted into an amount of species separation, which we define as the fraction of carbon atoms clustered in single-component regions. Here, we perform a linear interpolation between an expected value of *W*_R_ = 2.70 for completely mixed CH (no species separation) and *W*_R_ = 0.38 for fully demixed CH (100% species separation and the isolated carbon being in a diamond state) to obtain the corresponding values for the degree of species separation inside the sample. This procedure omits the first shock, however, DFT-MD simulations predict the Rayleigh scattering to be of very similar magnitude or slightly larger as for the double-shock conditions for mixed CH^45^. Moreover, the linear interpolation assumes demixing into pure constituents and no splitting into carbon-rich and hydrogen-rich phases in the regions where phase separation occurs. This assumption is consistent with previously published XRD data showing diamond formation and the remaining liquid structure well matched with a 1:1 CH mixture^28^. In case of demixing into significantly non-pure constituents, e.g. C_2_H + CH_2_, the inferred demixing would be larger due to a non-linear scaling of *W*_R_ for this case^45^.

**Fig. 4:**
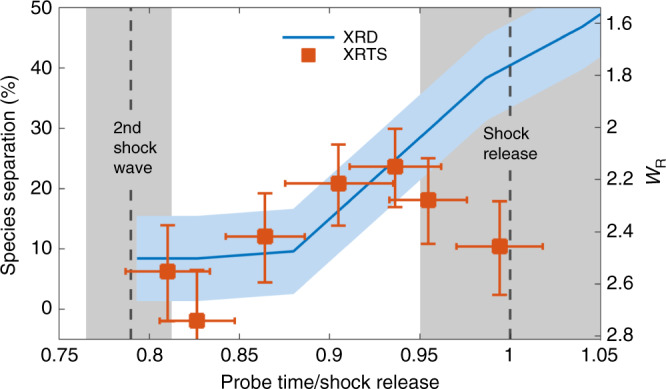
**C-H species separation results**. The measured Rayleigh scattering in forward direction ${W}_{{\rm{R}}}^{{\rm{f}}}$ is converted into a degree of species separation using DFT-MD simulations from Fig. 3. The probe time of each data point is normalized to the individually determined shock breakout time. The dashed vertical lines mark the start of the second shock wave and the onset of the shock release, respectively. The gray areas illustrate the corresponding temporal uncertainties. As the second shock propagates, the observed degree of species separation is in good agreement with diffraction data taken in the same experiment. The light blue area ilustrates the uncertainty of the diffraction measurements and the orange error bars depict the combined errors of timing and Rayleigh scattering intensity.

The results obtained by the procedure above are shown in Fig. 4. Once the second shock wave enters the target material, carbon and hydrogen start to separate from each other. With the progression of the second shock wave, the degree of species separation is increasing up to a maximum value of $\left(2{4}_{-7}^{+6}\right)$%. The later timings shown in Fig. 4 start to overlap with the shock release, where the rapid decompression leads to a significant signal increase at low *k*, which therefore obscures the inferred demixing. In particular, the liquid peak of the compressed mixture at  ~3 Å^−1^ broadens and shifts once the release starts^15^. Thus, the depicted data and error bars generally relate to the Rayleigh weight *W*_R_ and can only directly be connected to species separation as along as the shock waves are traveling inside the sample.

## Discussion

The observed results are in good agreement with XRD data recorded in the same experiment which show the formation of nanodiamonds induced by the second shock wave^15,23^. In contrast to the diffraction method, the absolute values for the C–H separation obtained by XRTS do not rely on the formation of crystalline structures in the experiment. However, the close match with XRD indeed suggests that the pure carbon clusters nearly exclusively consist of diamond, which is not embedded in a significant amount of liquid carbon. Assuming the carbon clusters consisting of only liquid carbon would lead to slightly larger values (~28%) for the degree of species separation inferred from XRTS due to the difference of *W*_R_ at the wavenumber of the FXRTS diagnostics (cf. Fig. 3).

Our measurements and its analysis also indicate that for a generalized use of this method, the boundary conditions of mixed and demixed state need to be known to some extent to obtain quantitative results. This is particularly true for experiments where no additional XRD measurements are available. However, there exist many important cases where the boundary conditions seem very clear, e.g. hydrogen-helium demixing inside giant planets. Moreover, this method can be used for determining mixing on microscopic level of previously fully separated atomic species where the boundary conditions are again very well defined.

In conclusion, we have determined the degree of species separation as a function of time in a CH sample dynamically compressed to warm dense matter conditions from spectrally resolved X-ray scattering data obtained in forward and backward scattering geometry. These results are in good agreement with XRD data from the same sample. In this way, we demonstrate the capability of X-ray Thomson scattering to characterize dynamic miscibility properties of fluid mixtures. In subsequent experiments, these data can be used to provide access to diffusivity, the kinetics of chemical reactions or (de)mixing due to hydrodynamic instabilities in warm dense matter. In principle, the demonstrated method is therefore capable to shed light on the complex transitions of liquids deep inside giant planets, such as hydrogen-helium demixing as predicted to happen inside Saturn or, potentially, Jupiter. Moreover, our results also suggest that ablator-fuel mixing as observed in contemporary inertial confinement fusion schemes can quantitatively be investigated by X-ray scattering. Indeed, this is very practical since, for an appropriate choice of the investigated wavenumbers *k*, it is often much easier to field accurate XRTS measurements at two distinct values of *k* than XRD which needs to cover a broad *k*-range, in particular when investigating low-*Z* samples at optical laser-only facilities. Corresponding drive schemes at X-ray free electron lasers as well as the implementation of precise X-ray Thomson scattering at ICF facilities^46^ will soon allow for such measurements to be performed.

## Methods

### Experiment

The experiment was performed at the instrument for Matter in Extreme Conditions (MEC), an endstation of the Linac Coherent Light Source (LCLS) at the SLAC National Accelerator Laboratory^47,48^. The polystyrene samples had a thickness of 83.4 μm and were coated with 100 nm aluminum on the front and rear sides. The laser drive beams were each delivering up to 15 J in 10 ns at 527 nm with a focal spot of 200 μm in diameter using phase plates. The beams were combined and delayed relative to each other by 4.5 ns to achieve a step pulse laser profile on the target. The laser intensities of the two steps were (2.7 ± 0.25) TW/cm^2^ and (7.3 ± 0.5) TW/cm^2^, respectively. Shock dynamics and timing to realize coalescing shock waves at the sample’s rear side were adjusted and recorded by a Velocity Interferometer System for Any Reflector (VISAR)^49^, in some cases using LiF windows attached to the rear side of the polystyrene samples^15^. Each spectrometer uses a cylindrically bent Highly Annealed Pyrolytic Graphite (HAPG) crystal^50^ with a radius of curvature of 51.7 mm in von Hámos geometry coupled to a Cornell-SLAC Pixel Array Detector (CSPAD)^51^ to detect spectrally resolved X-rays. The relative sensitivity of both spectrometers was cross-calibrated by Ni-K_*β*_ emission at (8 264.78 ± 0.01) eV, which was detected simultaneously by both spectrometers. The signals of both spectrometers have been flat field corrected, eliminating any systematic energy-dependent influences on the signal. Additionally, contributions of unshocked material due to an early probe time have been removed by scaling remaining diffraction features of cold material to the previously collected scattering and diffraction from the ambient samples.

### Hydrodynamic simulations

The hydrodynamic simulation results using the code package HELIOS^52^ are based on the SESAME equation of state (EOS)^53^ table 7590 for polystyrene, which is strongly constrained on the Hugoniot curve by precision gas gun and laser shock experiments^54^. The drive intensities set in the simulations (2.25 and 6.7 TW/cm^2^ for the first and second step, respectively) have been adjusted to match the VISAR measurements and are in reasonable agreement with the experimental values. We find that the second shock wave enters the pre-compressed sample material at  ~6 ns and coalesces with the first compression wave at the target rear side at around 7.6 ns.

### DFT-MD simulations

All DFT-MD simulations were performed using the VASP package, version 5.2^33–36^. The electronic density in the simulation box with periodic boundary conditions was represented by a plane wave expansion with a cut-off energy of *E*_cut_ = 1000 eV. We used the Mermin formulation of DFT to optimize the Helmholtz free energy at a given temperature^55^. The electron–ion interaction was modeled using the projector augmented wave (PAW) approach, specifically the hard PAW pseudopotentials for carbon (four valence electrons, C_*h*_ and H_*h*_ from Feb 2004) and hydrogen as provided with VASP^37,56^. The exchange-correlation potential was taken in generalized gradient approximation in Perdew–Burke–Ernzerhof parametrisation (GGA-PBE)^38^. We generally sampled the Brillouin zone of the supercell at the *Γ*-point only. The electronic bands where populated using a Fermi distribution at the chosen temperature. We had to increase the number of computed electronic bands to at maximum 2000 in order to sample the tail of the Fermi distribution for all cases. The supercell contained approximately 500 atoms of carbon and hydrogen with the relevant stoichiometry, the movements of which were calculated using the Hellman–Feynman forces derived from the electron densities of DFT under the Born-Oppenheimer approximation. The time step was *t* = 0.2 fs and the DFT-MD run covered a time span of 4–20 ps. The ion temperature was controlled by a Nosé–Hoover thermostat^57^. From the recorded coordinates, the ion and electron structure, and therefore the intensity of the elastic X-ray scattering in this multi-component system, can be obtained^40^. In order to predict the effect of demixing on the ionic structure, and therefore on the X-ray scattering, we combined the outputs of simulations with different atomic ratios, weighted to give the correct overall atomic ratio. The inelastic scattering signal is dominated by the carbon component and we thus approximate it by the inelastic scattering signal of diamond. We use time-dependent density functional theory (TDDFT) computations for the inelastic scattering signal using a full-potential linearized augmented-plane wave code implemented in elk^58^. The bootstrap kernel was used in a TDDFT calculation using the Kohn–Sham orbitals applying PBE-GGA for the exchange-correlation potential. Bootstrap is a parameter free long-range exchange-correlation kernel implemented in elk, which has been shown to reproduce excitonic effects at a reasonable computational cost^59,60^. The TDDFT calculations were performed on a 20 × 20 × 20 **k**-point mesh and 16 bands in the unit cell at the experimentally obtained density of *ρ* = 4.1 g/cm^3^. TDDFT is capable of computing the response functions at finite momentum transfer. In order to choose the complementary **k** and **q**-points we use the relation ${n}_{{k}_{i}}\times {q}_{i}=N$ where ${n}_{{k}_{i}}$ is the number of **k**-points along **x**, *q*_*i*_ is the number of **q**-points along **x**, N is an integer which is also a factor of ${n}_{{k}_{i}}$ in accordance with the Monkhorst-Pack **k**-point-sampling^61^. For ${\bf{k}}=({N}_{{k}_{1}},{N}_{{k}_{2}},{N}_{{k}_{3}})$, we choose ${\bf{q}}=(\frac{i}{{N}_{{k}_{1}}},\frac{j}{{N}_{{k}_{2}}},\frac{k}{{N}_{{k}_{3}}})$ where *i*, *j*, *k* are integers^62^. This results in **q**_*i*_ = **q**_*j*_ = **q**_*k*_ and a smearing width of 0.01 a.u. is used to approximate the Dirac delta function. The Bootstrap kernel^59^, although expensive, is chosen due to the self-consistency procedure compared to the parameter dependent LRC kernel of Botti et al.^60,63^. Furthermore, the effect of the system size and the influence of the exchange-correlation kernel in TDDFT for diamond under high pressure and warm dense matter conditions is shown in the work of Ramakrishna et al.^64^. For the experimental conditions (*ρ* = 4.1 g/cm^3^), the imaginary part of the inverse dielectric function is more sensitive to the pressure/density than the temperature of the system^64^. An increase in pressure causes the band gap to increase and therefore the inelastic peak to red shift. On the other hand, the increasing temperature tends to close the band gap. In our case, the temperature effect is significantly smaller compared to the change induced by the elevated pressure. This is seen in the close resemblance of the plasmon peak locations obtained using TDDFT in the optical limit under ambient (~1.35–1.4 Ha at *ρ* = 4.1 g/cm^3^) and warm dense conditions (1.3 Ha at 150 GPa, 6000 K)^64^.

## Supplementary information

Supplementary Information

## Data Availability

The data that support the plots within this paper and other findings of this study are available from the corresponding author upon reasonable request. The raw data of the experiment are stored on tape (long term storage) at SLAC National Accelerator Laboratory. Processing and reduction of the raw data requires the computational services at SLAC.
