# Supplementary material for: Demonstration of X-ray Thomson scattering as diagnostics for miscibility in warm dense matter
Source: Nat Commun. 2020 May 26;11:2620. doi: 10.1038/s41467-020-16426-y (PMC7251136; doi:10.1038/s41467-020-16426-y)

Reviewers' comments:

Reviewer #1 (Remarks to the Author):

The authors demonstrate the use of XRTS to analyze the miscibility of carbon and hydrogen in warm dense conditions. The particular conditions investigated in this experiment are ones in which complementary XRD measurements were possible due to the formation of crystalline diamond. The previously published XRD results largely corroborate the inferences from XRTS. This is a valuable proof-of-principle and another novel use of XRTS that promises to be influential in this field. Indeed, the natural application for this technique is in studying the miscibility of mixtures in which none of the components separate into a crystalline phase. This paper is a nice step towards realizing that.

The central premise of this technique is the sensitivity of the Rayleigh scattering weight at small angles to whether the system under interrogation is mixed. This sensitivity is evident in DFT-MD calculations. The weight decreases as species separation increases and a linear relationship between the weight and species separation is assumed. This brings the weight and species separation into one-to-one correspondence. The premise is sound for the mixture under consideration. Thus the metrological advance that is necessary is a measurement of the forward Rayleigh scattering weight. This is achieved by measuring two XRTS spectra, one at the desired forward scattering angle and another at a backward scattering angle that is sufficient to separate the elastic and inelastic features. The backward scattering weight is obtained from atomic form factors and the measured ratio of the integrated elastic and inelastic scattering intensities. This is used as calibration for the forward scattering signal, the weight of which is related to the ratio of the forward to backward elastic scattering intensities and the measured backward scattering weight. There are also straight forward polarization-dependent factors and a factor accounting for the relative efficiency of the two detectors. Critically, the latter is calibrated independently using Ni K beta emission. The only missing piece is then the integrated forward elastic scattering intensity. This is obtained by subtracting the integrated forward inelastic scattering intensity computed using TDDFT from the measured forward scattering signal.

This technique is applied to the analysis of warm dense C-H mixtures created via laser-driven shocks in polystyrene at the MEC endstation at LCLS. The results agree reasonably well with XRD. Overall, the work is convincing and novel and I think the paper should be published in Nature Communications. However, there are a few points of elaboration/clarification that might make the publication stronger.

1.) The deviation between species separation inferred from the XRD and XRTS grows at the shock release. The authors indicate that the rapid decompression and strong signal increase distorts the inferred demixing. It would be useful to elaborate on why this is the case. Specifically, it isn't clear to me as to why this isn't reflected in larger error bars. If this really is strictly due to systematic error, what steps can be taken to mitigate this, if any? What ramifications will this have for measurements of other mixtures?

2.) TDDFT calculations are used to compute the integrated inelastic scattering component of the forward scattering spectrum, so that it can be subtracted off. These calculations were done on a unit cell of diamond-like carbon with the experimental density, rather than snapshots from the DFT-MD trajectory. Experientially/empirically, I expect that this is well-justified. It would be useful to briefly elaborate on why this is the case. It might also be useful to emphasize that the integrated inelastic intensity will be less sensitive to typical systematic errors in TDDFT / the extent to which you expect errors in TDDFT to impact the results.

3.) There seems to be a minor error in the DFT-MD Simulations portion of the Methods section. "The TDDFT calculations under adiabatic local density approximation (ALDA) for the xc kernel are performed using Bootstrap, a long range xc kernel implemented in elk..." The next sentence goes

on to indicate that the "PBE-GGA exchange correlation functional..." was used. There are three different approximations that are nominally being used - ALDA and Bootstrap approximations to the xc kernel, and the PBE-GGA approximation to the xc functional. I expect that only two are being used in practice. That is, the bootstrap and ALDA kernels are different approximations. I assume that the bootstrap kernel was used in a LR-TDDFT calculation in which the input Kohn-Sham response function was computed using orbitals determined in a self-consistent PBE-GGA calculation.

Best,
Andrew Baczewski

Reviewer #2 (Remarks to the Author):

High energy density experiments at XFEL sources are very challenging and research teams are to be applauded for undertaking them. Moreover, as the manuscript discusses, better understanding the behavior of materials at high energy densities is critical for improved understanding of the internal structure and dynamics of gas and ice giant planets and for improving prospects for ICF.

However, though the manuscript's abstract and introduction give the impression that XRTS provides a relatively unambiguous determination of miscibility, the method presented relies completely on comparison with simulation and it's this aspect that concerns me. How reliably do we know the accuracy of the simulated structure factors (somewhat addressed in reference 28) and, perhaps more importantly, how much will the actual interionic structure factors depend on the degree and nature of phase separation? As the manuscript says: "In case of demixing into significantly non-pure consituents, e.g. C2H + CH2, the inferred demixing would be larger due to a non-linear scaling of WR for this case." In this regard, Fig. 3 in Ref. 45 shows how much the simulated structure factor depends on the nature of demixing assumed. In the present manuscript's case, the authors have evidence from XRD that the phase separated carbon forms diamond crystals, but in using XRTS as the more general method that the manuscript envisions, this information would not be available. Thus, though the lower limit of 24% carbon atoms not being in solution with H might be a reasonable estimate in this case, can we expect that there wil be more ambiguity in the general case?

The authors deserve kudos for undertaking research in such a challenging area and bringing sophisticated simulations to bear. And it seems that XRTS can provide some information about phase separation. However, I worry that the intensity information from only two detectors leaves too much unknown about the details of the microscopic sample structure to enable a reliable quantitative determination of the degree of miscibility. I must admit that my knowledge of the high energy density field is limited, so that perhaps the information available will still be important.

We thank the referees for taking the time to assess our manuscript and providing very positive and constructive comments. We think that the revised manuscript gained significantly due to the referees' comments which are addressed in detail below.

Reviewer #1

The authors demonstrate the use of XRTS to analyze the miscibility of carbon and hydrogen in warm dense conditions. The particular conditions investigated in this experiment are ones in which complementary XRD measurements were possible due to the formation of crystalline diamond. The previously published XRD results largely corroborate the inferences from XRTS. This is a valuable proof-of-principle and another novel use of XRTS that promises to be influential in this field. Indeed, the natural application for this technique is in studying the miscibility of mixtures in which none of the components separate into a crystalline phase. This paper is a nice step towards realizing that.

The central premise of this technique is the sensitivity of the Rayleigh scattering weight at small angles to whether the system under interrogation is mixed. This sensitivity is evident in DFT-MD calculations. The weight decreases as species separation increases and a linear relationship between the weight and species separation is assumed. This brings the weight and species separation into one-to-one correspondence. The premise is sound for the mixture under consideration. Thus the metrological advance that is necessary is a measurement of the forward Rayleigh scattering weight. This is achieved by measuring two XRTS spectra, one at the desired forward scattering angle and another at a backward scattering angle that is sufficient to separate the elastic and inelastic features. The backward scattering weight is obtained from atomic form factors and the measured ratio of the integrated elastic and inelastic scattering intensities. This is used as calibration for the forward scattering signal, the weight of which is related to the ratio of the forward to backward elastic scattering intensities and the measured backward scattering weight. There are also straight forward polarization-dependent factors and a factor accounting for the relative efficiency of the two detectors. Critically, the latter is calibrated independently using Ni K beta emission. The only missing piece is then the integrated forward elastic scattering intensity. This is obtained by subtracting the integrated forward inelastic scattering intensity computed using TDDFT from the measured forward scattering signal.

This technique is applied to the analysis of warm dense C-H mixtures created via laser-driven shocks in polystyrene at the MEC endstation at LCLS. The results agree reasonably well with XRD. Overall, the work is convincing and novel and I think the paper should be published in Nature Communications However, there are a few points of elaboration/clarification that might make the publication stronger.

1.) The deviation between species separation inferred from the XRD and XRTS grows at the shock release. The authors indicate that the rapid decompression and strong signal increase distorts the inferred demixing. It would be useful to elaborate on why this is the case. Specifically, it isn't clear to me as to why this isn't reflected in larger error bars. If this really is strictly due to systematic error, what steps can be taken to mitigate this, if any? What ramifications will this have for measurements of other mixtures?

> We thank the referee for pointing out an important need for clarification. The error bars shown in Fig. 4 are specifically related to the measured Rayleigh weight W_R. The direct connection to C-H species separation relies on the assumption that the shock waves travel inside the sample. Once release starts at the rear side, rapid decompression significantly increases the Rayleigh Weight at small *k*. This is mainly due to the structure factor S_{CC} approaching unity for a disordered gas at low density. In turn, the broad liquid peak of the compressed mixture at ~3 Å^{-1} broadens and shifts to lower *k* once the release starts. This is well visible in our published diffraction data (see e.g. Fig. 3 of Nat. Astron. 1, 606-611 (2017)). With pressure release being present, it is of course no longer possible to directly relate values as well as errors of the Rayleigh weight to the inferred demixing. We have clarified the points discussed above in the text, which now reads:

"The later timings shown in Fig. 4 start to overlap with the shock release, where the rapid decompression leads to a significant signal increase at low k, which therefore obscures the inferred demixing. In particular, the liquid peak of the compressed mixture at ~3 Å^{-1} broadens and shifts once the release starts[15]. Thus, the depicted data and error bars generally relate to the Rayleigh weight W_R and can only directly be connected to species separation as along as the shock waves are travelling inside the sample."

2.) TDDFT calculations are used to compute the integrated inelastic scattering component of the forward scattering spectrum, so that it can be subtracted off. These calculations were done on a unit cell of diamond-like carbon with the experimental density, rather than snapshots from the DFT-MD trajectory. Experientially/empirically, I expect that this is well-justified. It would be useful to briefly elaborate on why this is the case. It might also be useful to emphasize that the integrated inelastic intensity will be less sensitive to typical systematic errors in TDDFT / the extent to which you expect errors in TDDFT to impact the results.

Indeed, we think that using diamond instead of a DFT-MD snapshot is justified and practical since the inelastic signal is mostly dominated by C in form of diamond. Moreover, we cannot do TDDFT on an original snapshot with N=500 atoms overall.

We have added some clarifying text to the Methods Section:

"The Bootstrap kernel[59,] although expensive, is chosen due to the self-consistency procedure compared to the parameter dependent LRC kernel of Botti et al.[60,63]. Furthermore, the effect of the system size and the influence of the exchange-correlation kernel in TDDFT for diamond under high pressure and warm dense matter conditions is shown in the work of Ramakrishna et al.[64]. For the experimental conditions (ρ=4.1 g/cm^3), the imaginary part of the inverse dielectric function is more sensitive to the pressure/density than the temperature of the system[64]. An increase in pressure causes the band gap to increase and therefore the inelastic peak to red shift. On the other hand, the increasing temperature tends to close the band gap. In our case, the temperature effect is significantly smaller compared to the change induced by the elevated pressure. This is seen in the close resemblance of the plasmon peak locations under ambient (~1.35-1.4 Ha at ρ=4.1 g/cm3) and warm dense conditions (1.3 Ha at 150 GPa, 6000 K)[64]."

3.) There seems to be a minor error in the DFT-MD Simulations portion of the Methods section. "The TDDFT calculations under adiabatic local density approximation (ALDA) for the xc kernel are performed using Bootstrap, a long range xc kernel implemented in elk..."

We thank the referee for pointing this out. Indeed, this was a mistake. We have corrected the corresponding sentences to:

"We use time-dependent density functional theory (TDDFT) computations for the inelastic scattering signal using a full-potential linearised augmented-plane wave code implemented in elk[58]. The bootstrap kernel was used in a TDDFT calculation using the Kohn-Sham orbitals applying PBE-GGA for the exchange-correlation potential. Bootstrap is a parameter free long-range exchange-correlation kernel implemented in elk, which has been shown to reproduce excitonic effects at a reasonable computational cost[59,60]. The TDDFT calculations were performed on a 20 x 20 x 20 **k**-point mesh and 16 bands in the unit cell at the experimentally obtained density of ρ=4.1 g/cm^3."

Reviewer #2:

High energy density experiments at XFEL sources are very challenging and research teams are to be applauded for undertaking them. Moreover, as the manuscript discusses, better understanding the behavior of materials at high energy densities is critical for improved understanding of the internal structure and dynamics of gas and ice giant planets and for improving prospects for ICF.

However, though the manuscript's abstract and introduction give the impression that XRTS provides a relatively unambiguous determination of miscibility, the method presented relies completely on comparison with simulation and it's this aspect that concerns me. How reliably do we know the accuracy of the simulated structure factors (somewhat addressed in reference 28) and, perhaps more importantly, how much will the actual interionic structure factors depend on the degree and nature of phase separation? As the manuscript says: "In case of demixing into significantly non-pure consituents, e.g. C2H + CH2, the inferred demixing would be larger due to a non-linear scaling of WR for this case." In this regard, Fig. 3 in Ref. 45 shows how much the simulated structure factor depends on the nature of demixing assumed. In the present manuscript's case, the authors have evidence from XRD that the phase separated carbon forms diamond crystals, but in using XRTS as the more general method that the manuscript envisions, this information would not be available. Thus, though the lower limit of 24% carbon atoms not being in solution with H might be a reasonable estimate in this case, can we expect that there will be more ambiguity in the general case?

> We thank the referee for pointing out an important issue that requires clarification. Certainly, our measurement crucially relies on the calculated structure factors. Nevertheless, DFT-MD is the best method currently available for the investigated conditions and has shown extraordinarily good agreement in many experiments studying the warm dense matter regime (see e.g. L. B. Fletcher et al., Nature Photonics 9, 274-279 (2015)).
>
> Moreover, we agree that quantitative interpretation of (de)mixing can require assumptions on the underlying process and the final products. However, in many cases, like H-He separation, where full separation is expected for certain planetary interior conditions, these boundary conditions are very clear as shown by theoretical feasibility studies on the sensitivity of XRTS (K. Wünsch et al., Ref 24). On the other hand, it should be noted that the presented method is not only capable of determining demixing of previously mixed species, but also the other way around: mixing on microscopic level of previously separated atomic species. The latter is highly important for, e.g., inertial confinement fusion implosions, but also a promising way to measure transport, in particular diffusivity, at extreme pressure and temperature conditions. In this case, the boundary condition of fully separated atomic species as initial state is very clear and, independently on the specific mixing process, our method is highly sensitive to mixing on the microscopic level, which is, to our best knowledge, very difficult to obtain by any other diagnostics in this regime.
>
> This is an important point that apparently was not stressed enough in the original version of the manuscript. We have now adjusted the text accordingly:
>
> "Our measurements also indicate that for a generalized use of this method, the boundary conditions of mixed and demixed state need to be known to some extent to obtain quantitative results. This is particularly true for experiments where no additional XRD measurement is available. However, there exist many cases where the boundary conditions seem very clear, e.g. hydrogen-helium demixing inside giant planets. Moreover, this method can be used for determining mixing on microscopic level of previously fully separated atomic species."

The authors deserve kudos for undertaking research in such a challenging area and bringing sophisticated simulations to bear. And it seems that XRTS can provide some information about phase separation. However, I worry that the intensity information from only two detectors leaves too much unknown about the details of the microscopic sample structure to enable a reliable quantitative determination of the degree of miscibility. I must admit that my knowledge of the high energy density field is limited, so that perhaps the information available will still be important.

> We thank referee for acknowledging the significant effort connected with our studies. The astonishing developments in experimental infrastructures and computational methods now enable such studies. Answering the specific question, we think that for a clever choice of the wavenumber *k*, two detectors are indeed sufficient for several interesting cases with clear

boundary conditions (as discussed above). This is an important feature and will enable the application at high energy laser facilities without XFEL source, where a full *k*-resolved measurement of the structure factor is difficult to achieve.

We adjusted a corresponding sentence in the final paragraph of the main text:

"Indeed, this is very practical since, for an appropriate choice of the investigated wavenumbers *k*, it is often much easier to field accurate XRTS measurements at two distinct values of *k* than XRD which needs to cover a broad k-range, in particular when investigating low-Z samples at optical laser-only facilities."

REVIEWERS' COMMENTS

Reviewer #1 (Remarks to the Author):

The authors have adequately addressed my comments. Their edits and additions have improved the quality of an already well-composed manuscript. I recommend publication.

Reviewer #2 (Remarks to the Author):

The authors' changes have addressed the concerns that I previously had about the manuscript. The approach pioneered here appears to be well positioned to contribute important new information to our understanding of highly compressed material at high temperatures.

One very small point the authors may wish to look at is that, on page 8 of the manuscript, it says that WR = 2.70 for completely mixed CH. However, as best as I can tell from Fig. 3, WR for mixed CH seems slightly lower than this.

We thank the referees for again assessing our manuscript and their recommendation of publication. Please find below a response to referee 2's remaining comment.

> *One very small point the authors may wish to look at is that, on page 8 of the manuscript, it says that WR = 2.70 for completely mixed CH. However, as best as I can tell from Fig. 3, WR for mixed CH seems slightly lower than this.*

We thank the referee for finding a small but important mistake. Indeed Figure 3 was showing a DFT-MD curve for 6000 K instead of 5000 K due to a typo in the plotting script. We changed the curve to the correct one in the current version of the manuscript. As seen below in the plot showing both curves, the differences are very subtle, however, now everything is consistent with the plot showing W_R ~2.7 for the FXRTS as is correctly used for the calculations throughout the manuscript.

The 5000 K curve is consistent with hydro simulations and was previously shown to best match the diffraction data taken simultaneously in the experiment (D. Kraus et al., Phys. Plasmas 25 056313 (2018)).

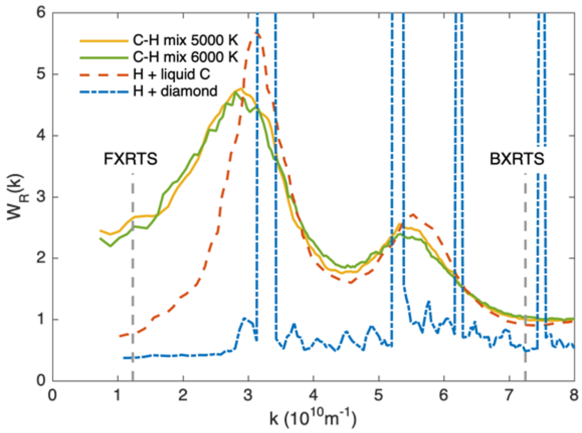

Supplement: Supplementary file 1 — Supplementary Information [file 41467_2020_16426_MOESM1_ESM.pdf]
